# Proposal of a new equation for estimating resting energy expenditure of acute kidney injury patients on dialysis: a machine learning approach

**DOI:** 10.1186/s12986-020-00519-y

**Published:** 2020-11-17

**Authors:** Daniela Ponce, Cassiana Regina de Goes, Luis Gustavo Modelli de Andrade

**Affiliations:** Department of Internal Medicine - UNESP, Univ Estadual Paulista, Rubião Jr, s/n – Botucatu/SP18.618-970, São Paulo, Brazil

**Keywords:** Energy metabolism, Resting energy expenditure, Machine learning, Acute kidney injury, Sepsis, Dialysis

## Abstract

**Background:**

The objective of this study was to develop a new predictive equation of resting energy expenditure (REE) for acute kidney injury patients (AKI) on dialysis.

**Materials and methods:**

A cross-sectional descriptive study was carried out of 114 AKI patients, consecutively selected, on dialysis and mechanical ventilation, aged between 19 and 95 years. For construction of the predictive model, 80% of cases were randomly separated to training and 20% of unused cases to validation. Several machine learning models were tested in the training data: linear regression with stepwise, rpart, support vector machine with radial kernel, generalised boosting machine and random forest. The models were selected by ten-fold cross-validation and the performances evaluated by the root mean square error.

**Results:**

There were 364 indirect calorimetry measurements in 114 patients, mean age of 60.65 ± 16.9 years and 68.4% were males. The average REE was 2081 ± 645 kcal. REE was positively correlated with C-reactive protein, minute volume (MV), expiratory positive airway pressure, serum urea, body mass index and inversely with age. The principal variables included in the selected model were age, body mass index, use of vasopressors, expiratory positive airway pressure, MV, C-reactive protein, temperature and serum urea. The final r-value in the validation set was 0.69.

**Conclusion:**

We propose a new predictive equation for estimating the REE of AKI patients on dialysis that uses a non-linear approach with better performance than actual models.

## Introduction

Acute kidney injury (AKI) occurs in approximately 3–15% of hospitalised patients and can affect 30–50% of patients admitted to intensive care units (ICU). It is associated with extremely high mortality rates, ranging from 20 to 50% [1]. Previous observational studies reported that malnourished and hospitalised AKI patients have higher rates of morbidity and mortality than well-nourished patients [2, 3] and an association between cumulative caloric deficits and poor outcome in ICU patients [4, 5]. Accurate determination of energy needs is obviously important in critically ill patients as both over and underfeeding may be associated with complications and undesired consequences [6].

Determining energy requirements in critically ill patients via indirect calorimetry (IC) has long been considered the gold standard [7]. Limitations for using IC include time constraints, equipment availability, staffing and cost. Therefore, many predictive equations exist for predicting resting energy expenditure (REE), but the accuracy of these equations for estimating caloric requirements of critically ill patients is unclear [7–13]. Goes et al. evaluated if nine different standard predictive equations for energy expenditure could accurately reflect the energy requirements of critically ill, mechanically ventilated AKI patients [14]. There was low precision and poor agreement between measured and predicted REE by the Harris–Benedict (HB), Mifflin, Ireton–Jones, Penn State, American College of Chest Physicians and Faisy equations. The HB, without using the injury factor, was the least precise (18% precision). Modified Penn State equation had the best precision, although the precision rate was only 41%. In conclusion, none of these equations accurately estimated measured REE in severe AKI patients on dialysis and most of them underestimated energy needs [14]. Recently more sophisticated models using machine learning were applied in clinical practices resulting in better predictive models [15]. These models can be applied to build predictive equations aiming to capture the non-linearity between variables, resulting in better accuracy. These new models have also been applied to better predict resting energy expenditure (REE) [16].

The present study aimed, therefore, to develop a new predictive equation of REE for AKI patients, on dialysis and mechanically ventilated, using a machine learning approach.

## Materials and methods

A cross-sectional descriptive study was carried out in the Dialysis Unit of the Clinical Hospital of Botucatu Medical School. The project was approved by the centre’s ethical committee and the protocols used followed the criteria of the Helsinki Declaration (protocol 4383/2012). For development of the predictive equation, 364 indirect calorimetry (IC) measurements in 114 AKI patients on dialysis were consecutively selected, aged between 19 and 95 years. Subjects with a body mass index (BMI) between 18–55 kg/m^2^, who gave their consent to participate in the study and agreed to comply with its standards were included. Inclusion criteria were patients admitted to the ICU with a diagnosis of AKI according to the KDIGO criteria [17], clinical symptoms suggestive of sepsis and acute tubular necrosis (ATN), a need for renal replacement therapy (RRT) (stage 3) and mechanically ventilated. Exclusion criteria were patients with AKI of other aetiologies, renal transplanted or those with chronic renal disease stages 4 and 5 (glomerular filtration rate (GFR) < 30 ml/min estimated by the Modification of Diet in Renal Disease (MDRD) equation) [18], a fraction of inspired oxygen (FiO_2_) greater than 0.60, expiratory positive airway pressure (PEEP) > 10 cm H_2_O, maximum airway pressure > 60 cm H_2_O, stirring presence, use of neuromuscular blockers, air leakage into the ventilator circuit around the endotracheal tube cuff or from a bronchopleural fistula, as these factors lead to inaccuracies in REE measurement by IC. IC was performed using the RMR Quark apparatus (Cosmed, Rome, Italy).

### Data analysis

**Fig. 1:**
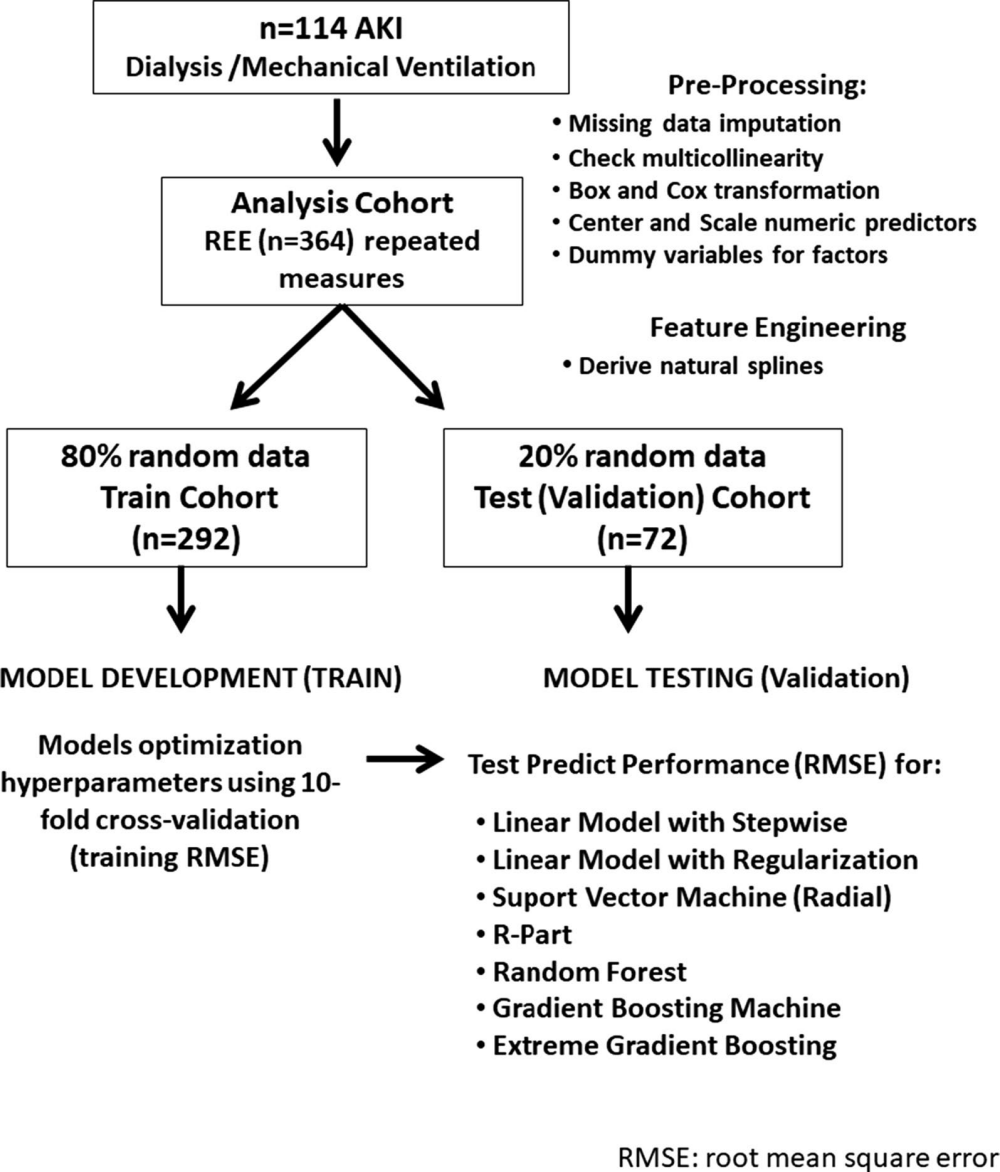
Flowchart of the study design and description of the machine learning approach

For construction of the predictive model, 80% of the cases were randomly separated. Twenty per cent of unused cases were separated for validation. The pre-processing was applied to the training and test set. The continuous predictor variables were transformed using Box and Cox and after dividing by means (centre) and standard deviation (scale). This transformation led to a uniform scale (mean = 0, SD = 1) for all analyses so that they were comparable between analytical platforms. The missing variables were imputed by the median. Categorical variables were converted into dummy variables. In feature engineering, we tested the distribution of each continuous variable according to the outcome. We choose natural splines with three degrees of freedom for age and BMI aims to account for the non-linearity. Afterward, linear and non-linear models were tested in the training data. These models were: linear regression with stepwise selection, linear regression with regularisation (glmnet), rpart, support vector machine with radial kernel (SVM), generalised boosting machine (GBM), extreme gradient boosting (XGBoost) and random forest. The optimisation of hyperparameters was done in the training set with ten-fold cross-validation and the performance was evaluated by the root mean square error (RMSE). Therefore, we evaluated the final performance of the best model in the test set by choosing the model with the lower RMSE (Fig. 1). The analysis was performed with R version 3.6.3, Vienna Austria, 2020 with caret package.

## Results

A total of 364 IC measures in 114 patients were evaluated. The mean age was 60.65 ± 16.9 years and 68.4% were males. Diagnoses of sepsis and cardiovascular disease (CVD) accounted for 79.8% of hospitalisations. AKI was the aetiology associated with sepsis in most patients (81.6%). The REE estimated by the Harris–Benedict (HB) formula averaged 1540 ± 346 kcal; whereas the REE measured by the IC was significantly higher 2081 ± 645 kcal (27.7 ± 10 kcal/kg/day, *p* < 0.001). Table 1 shows the clinical characteristics of the general population studied at the time when dialysis was indicated.

**Table 1:** Clinical characteristics of the population with AKI on dialysis

*Patients parameters* (n = 114)	
Age (years)	60.6 ± 16.9
Male gender (%)	78 (68.4)
AKI (%)	
Sepsis-related	93 (81.6)
ischemic	13 (11.4)
Nephrotoxic	6 (5.3)
Mixed	2 (1.8)
ATN-ISS	0.64 ± 0.18
Race (%)	
White	98 (86)
Black	11 (9.6)
Weight (kg)	77.6 ± 22.4
Main diagnosis	
CVD	35 (30.7)
Sepsis, severe sepsis, shock	56 (49.1)
Neoplasia	9 (7.9)
Liver diseases	8 (7)
Trauma	6 (5.3)
Death	73 (63)
*Patients and IC parameters* (n = 364)	
REE using HB (Kcal)	1540 ± 346
REE using IC (Kcal)	2081 ± 645
REE using IC (Kcal/kg/d)	27.9 ± 10.4
VAD (mcg/kg/min)	0.11
	(0.00–0.35)
MV	8.5 ± 2.8
Freq. (resp/min)	17 ± 5.2
PEEP (cm H_2_O)	6 ± 1.5
FIO_2_ (%)	38 ± 10.9
Temperature (°C)	37.6 ± 0.8
Urea (mg/dl)	157 ± 73
WBC (mm^3^)	17,300 (12,300–25,500)
PCR (mg/dl)	23.1 (7.6–32.8)

*CVD* cardiovascular disease, *AKI* acute kidney injury, *ATN-ISS* individual severity score in acute tubular necrosis, *REE* resting energy expenditure, *VAD* vasoactive drug, FIO_2_ fraction of inspired oxygen, *Mv* minute volume, *RR* respiratory rate, *PEEP* expiratory positive airway pressure, *Creat* serum creatinine, *WBC* white blood-cell count, *CRP* C-reactive protein

**Fig. 2:**
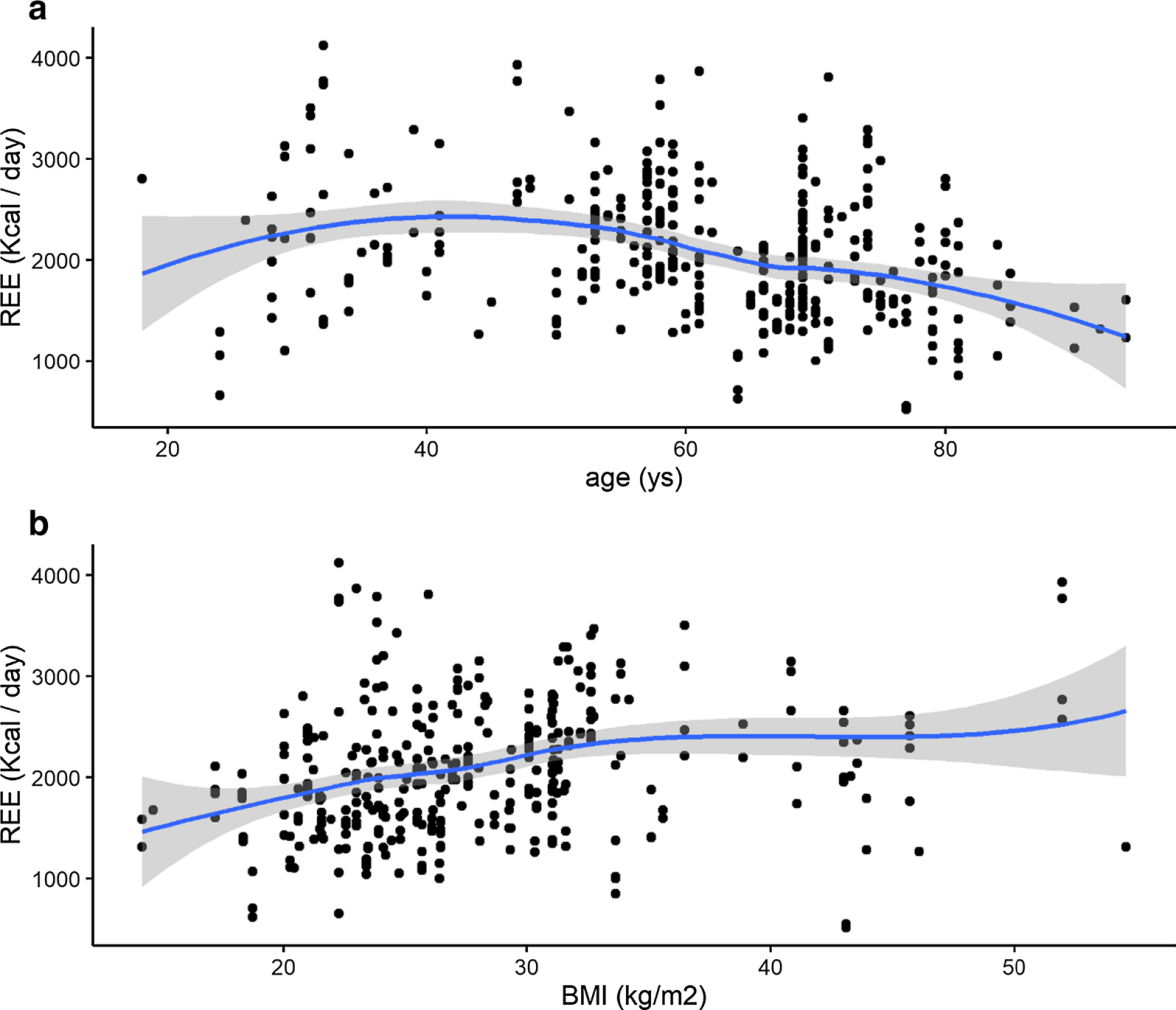
A plot of age-related to REE (**a**) and BMI related to REE (**b**). The blue line is a smooth line that was fitted using polymonial regression

The variables available to train the models of REE in the total population (n = 364) are summarised in attachment 01. In the exploratory data analysis, we plotted the REE related to age and BMI (Fig. 2). We demonstrated a non-linear association between these predictors and the outcome. Then, we applied natural splines with three degrees of freedom aiming to capture the non-linearity (feature engineering).

**Fig. 3:**
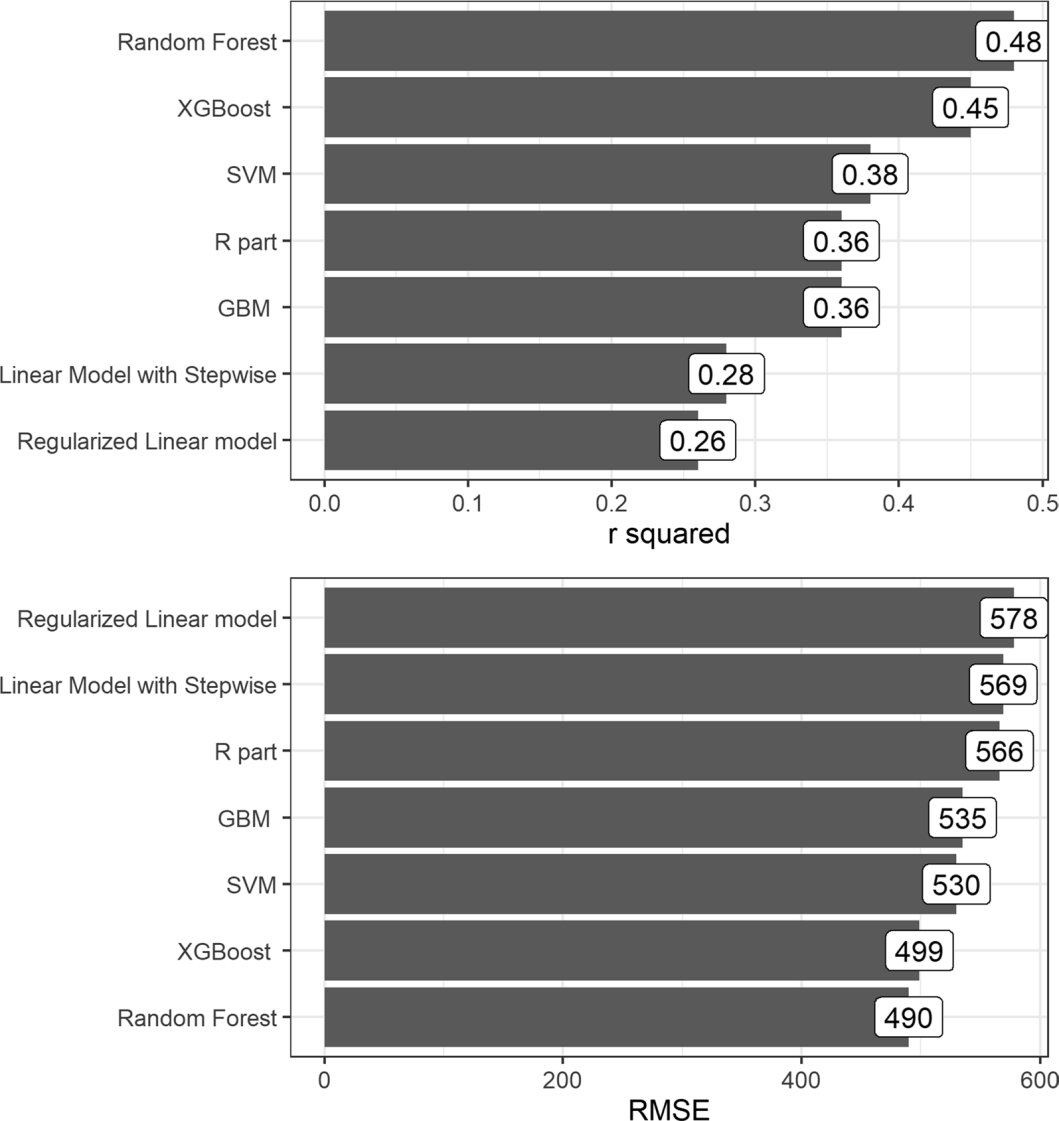
Summary statistics of tuned trained models in resamples of the training set. The box summaries the mean values. The statistics were: R squared and root mean square error (RMSE)

We trained different algorithms by ten-fold cross-validation in the training set (80% of the data) and the hyperparameters of each model were tuned based in the lower RMSE. After training tuned models, we applied resamples of the training data and selected the best model based on the lower RMSE (Fig. 3). Therefore, the final models were evaluated for performance in the test set (internal validation) aiming to confirm the results of the training set (Table 2).

**Table 2:** Summary statistics of final tuned models in the test set (20% of the data). R^2^ (R squared), and root mean square error (RMSE)

Models	R^2^	RMSE
Linear model with stepwise	0.28	569.24
Regularized linear model	0.26	578.44
SVM	0.38	530.05
Random forest	0.48	490.05
R part	0.36	566.61
GBM	0.36	535.88
XGBoost	0.45	499.75

**Fig. 4:**
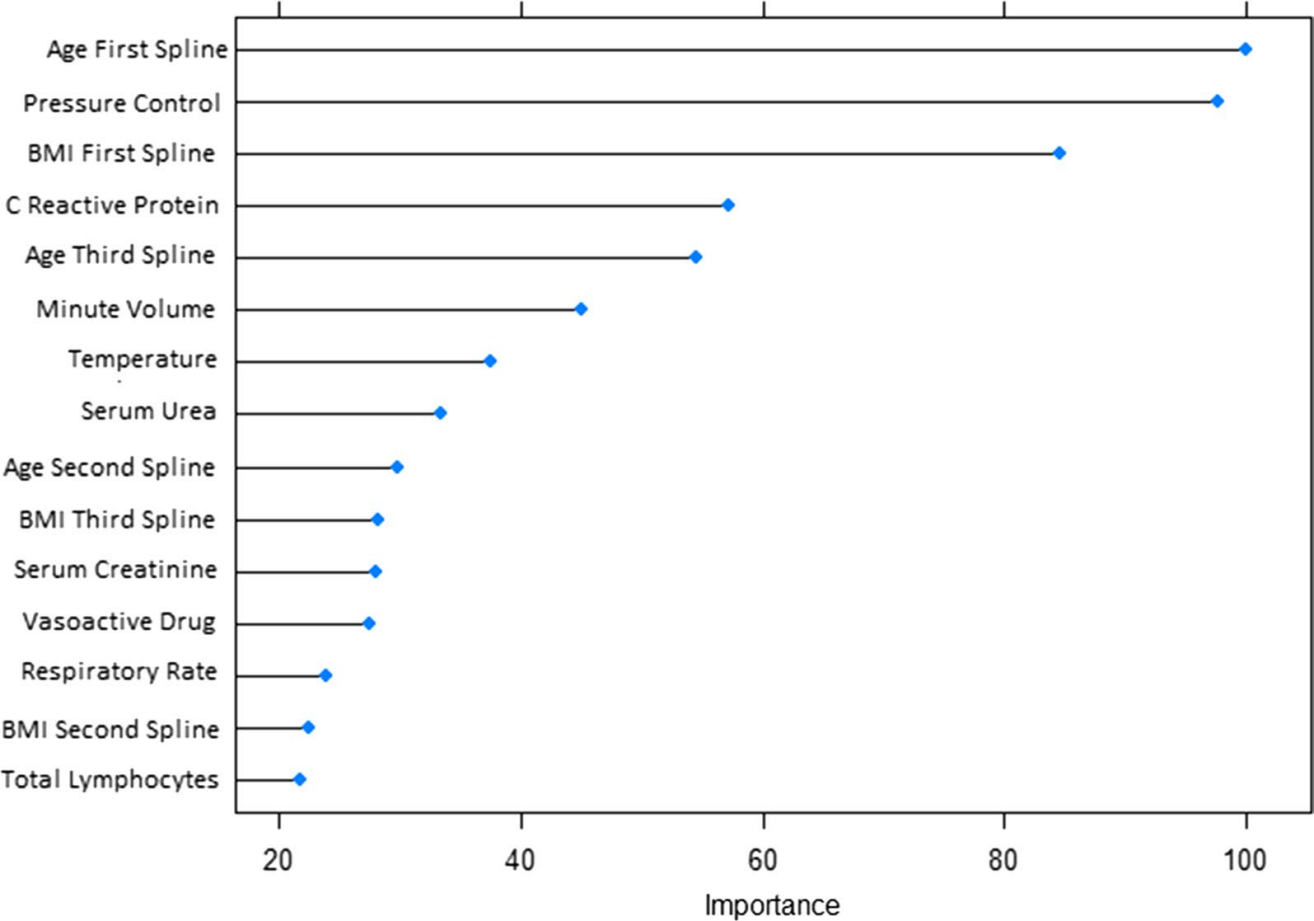
Variable importance in the random forest model. The importance was reported on a normalised scale

The models that had a non-linear approach had a lower RMSE. The linear model may have had the advantage of simplicity and interpretability (attachment 02). The model with the best accuracy was selected by the lower RMSE: random forest. The variable importance of the final model (random forest) was plotted in Fig. 4.

**Fig. 5:**
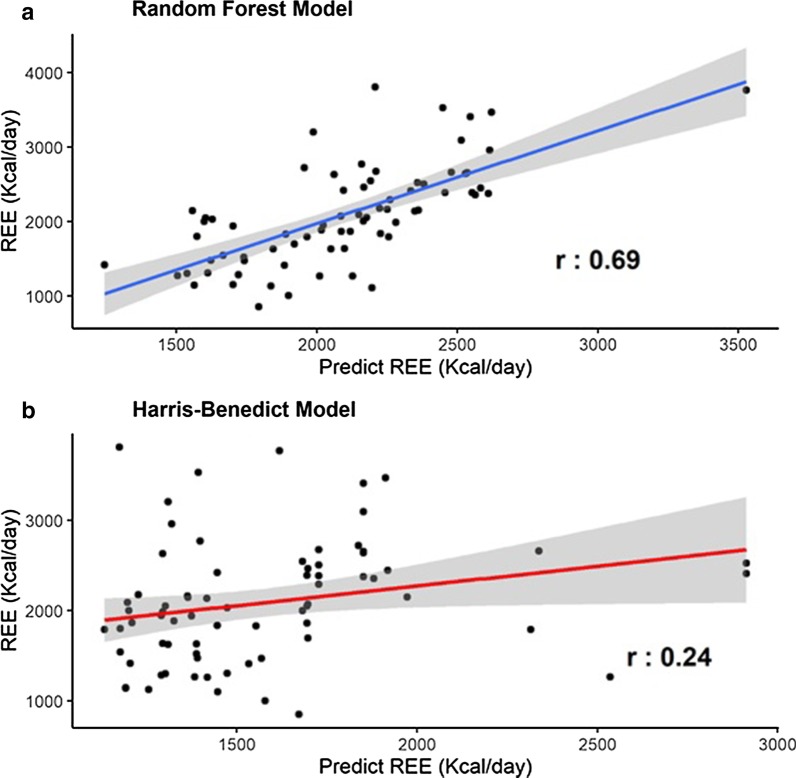
A correlation plot between the predicted and observed REE in the test set (20% of unseen data). **a** Predicted REE by random forest model and **b** predicted REE by Harris–Benedict equation. We had an r-value of 0.69 with the random forest model (new model) and an r-value of 0.24 with the Harris–Benedict

Finally, we plotted a correlation between the predicted values of REE and observed REE in the test set (unseen data). The random forest model (best model) had a final r-value in this test set of 0.69 compared to an r-value of 0.24 with the Harris–Benedict equation (Fig. 5).

## Discussion

Information about the energy expenditure assessment of patients with AKI is scarce in the literature. Our study found that the REE estimated by the Harris and Benedict formula [19] was significantly lower than that measured by IC. This finding corroborates the indication not to use this formula in critically ill patients and in patients with AKI [11, 14, 20, 21] and the need to propose a new equation for those AKI on dialysis [21–25].

It has long been held that critical illness is a hypermetabolic state, i.e. that the basal metabolic rate is higher than that predicted by simple population characteristics [25–27]. Physiological factors such as fever, increased substrate cycling, and synthetic functions associated with the host response to stress and inflammation provide a theoretical basis for this generalization. Energy expenditure is also influenced by common ICU therapies [14, 25].

This study aimed to develop and validate predictive equations for REE in severe AKI patients using a machine learning approach. It was found that the models were developed validly and significantly predicted REE in these patients and according to several linear and non-linear algorithms. In the present study, REE was positively correlated with C-reactive protein, minute volume (MV), expiratory positive airway pressure, serum urea, BMI and inversely with age (attachment 02). The principal variables included in the best model were age, BMI, use of vasopressors, expiratory positive airway pressure, minute volume, C-reactive protein, temperature and serum urea. The final r-value in the validation set was 0.69. In the literature, there is no consensus regarding the procedures to be used for the validation of predictive models. Some authors do not suggest the use of determination and correlation coefficients for the validation of techniques or estimated variables [15]. Others consider that the Bland Altman plot is likely to show a systematic proportion bias [19]. We used ten-fold cross-validation and selected the model with the lower RMSE. The performance of the best model was confirmed in a test set of 20% randomly selected unseen data (internal validation). The linear models had the advantage of simplicity of the model built and better interpretability but did not capture the non-linearity of the data. We confirmed this with a very low accuracy of the Harris–Benedict equation in the test set. The use of natural splines to age and BMI predictors improved the linear models but did not reach the same performance as the non-linear models like boost trees or support vector machines [22, 23]. The best model had a higher performance but a trade-off in lower interpretability. In addition, the traditional model like the Harris–Benedict [19] uses linear models that were easy to implement. Otherwise, the implementation of a model, like the random forest, that requires a calculator. However, currently, we have the facility of computers or apps that may overcome this difficulty.

Another advantage of the machine learning approach is to demonstrate other predictor variables that influence the outcome by finding complex interactions. The principal factors that contributed to the variability of REE were the BMI and age that were previously described with traditional models [24, 25]. The machine analysis in this study reveals other new contributary variables that predict the REE, for example, ventilator parameters and biochemical values. Otherwise, a simple linear model with this data set without pre-processing and feature engineering results in a model with lower accuracy and does not shown new interactions (R^2^ = 0.21, data not shown).

Thus, this study demonstrates the importance of estimating the REE in severe AKI patients, which is a determining factor in the assessment of nutrition status in critically ill patients. Previous studies reported correlations between morbidity or mortality and REE and indicated that sicker patients and non-survivors had a lower REE [22, 25] but this is not a universal observation [26, 27]. Interpretations of these findings are limited by potential survivorship bias and confounders such as age and physical activity.

From a practical and application point of view, it is worth mentioning the capacity for technical evaluations to reduce the difference in REE between IC and that estimated by other conventional formulas, such as HB, the use of these predictive equations for the assessment of passive and active trawling, contributing with relevant information for nutritionists and physicians.

The present study has as main limitations the fact that the predictive models are not valid for non- severe AKI patients and the Bland Altman model was not performed. Although we trained models with a robust estimator with ten-fold cross-validation using RMSE as a metric, an approach regularly used in machine learning analysis, we tested the model in unseen data that may be considered an internal validation.

## Conclusion

We propose a new predictive equation for estimating the REE of AKI patients on dialysis that uses a non-linear approach with better performance than actual models.

## Data Availability

All data generated or analysed during this study can be included in this published article as supplementary information files.
